# Non-Diffractive Beams for State-of-the-Art Applications

**DOI:** 10.3390/mi15060771

**Published:** 2024-06-09

**Authors:** Muhammad A. Butt, Svetlana N. Khonina

**Affiliations:** Samara National Research University, 443086 Samara, Russia

Non-diffractive beams, also known as diffraction-free beams, are a class of optical beams that maintain their intensity profile over a long distance without spreading out due to diffraction [1,2,3]. There are several types of non-diffractive beams, each with unique properties and a range of applications across various fields. Bessel beams are perhaps the most well-known type of non-diffractive beams [4]. They are characterized by their ability to reconstruct themselves after encountering an obstacle, a property known as self-healing. The electric field distribution of an ideal Bessel beam is given by a Bessel function of the first kind. These beams can be generated using axicons, which are conical lenses that transform a Gaussian beam into a Bessel beam. In microscopy, Bessel beams are used to enhance imaging depth and resolution. Their self-healing properties enable imaging through turbid media, making them ideal for biological imaging [5]. Bessel beams can also be used to trap and manipulate microscopic particles. Their extended depth of focus allows for three-dimensional control in optical tweezers [6]. The high-intensity central core of Bessel beams is useful in precision laser machining and materials processing, enabling fine cuts and modifications at micron scales.

Airy beams are another type of non-diffractive beams known for their unique parabolic trajectory and self-healing properties [7]. Unlike Bessel beams, Airy beams exhibit an intensity profile that accelerates during propagation. They are generated by modulating the phase of a Gaussian beam with a cubic phase mask. Airy beams are used in optical trapping to move and guide particles along curved paths [8]. This capability is beneficial in microfluidic devices for the precise control of particle trajectories. The ability of Airy beams to propagate along curved paths makes them useful in medical imaging and targeted therapy, especially in navigating obstacles within biological tissues. In light-sheet microscopy, Airy beams provide an enhanced field of view and improved imaging of dynamic biological processes due to their extended depth of focus and curved propagation path.

Mathieu beams are a type of non-diffractive beams with elliptical symmetry, described mathematically by Mathieu functions [9]. These beams can propagate without changing their intensity distribution and exhibit a unique helical or elliptical intensity pattern. Mathieu beams are used in quantum optics experiments due to their unique phase and intensity distributions. They can be used to manipulate the quantum states of light and for precision measurements [10]. In advanced microscopy techniques, Mathieu beams can improve the imaging resolution and contrast, especially in complex biological samples where traditional beams may struggle.

Vortex beams, or optical vortices, possess a phase singularity and carry orbital angular momentum [11]. Phase singularity causes the beam to have a doughnut-shaped intensity profile with a dark central region. Vortex beams are utilized in free-space optical communication systems to increase data transmission rates by encoding information in orbital angular momentum states. The angular momentum of vortex beams is employed in optical tweezers to rotate and manipulate particles, which is valuable in biological and materials science research [12]. In quantum computing and information, vortex beams are used to manipulate and encode information in the orbital angular momentum states of photons, enabling advanced quantum communication protocols. In summary, non-diffractive beams offer numerous advantages in precision, control, and resolution across various scientific and industrial applications. Their ability to maintain their shape and intensity over long distances, as well as their unique properties such as self-healing and orbital angular momentum, make them indispensable tools in modern optics and photonics.

This Special Issue “Non-Diffractive Beams for State-of-the-Art Applications” comprises a total of 12 papers, among which 10 contribute original research findings on non-diffractive beams, while the remaining 2 offer comprehensive reviews. Each of these papers delves into diverse aspects of their respective fields, presenting novel insights, methodologies, and discoveries. From pioneering experimental studies to theoretical explorations, the range of topics covered in this collection reflects the dynamic breadth of contemporary research endeavors. The original research works offer fresh perspectives and innovative approaches to addressing complex challenges, contributing significantly to the advancement of knowledge in the domain of non-diffractive beams. Meanwhile, the review papers provide valuable synthesis and critical analysis of the existing literature on non-diffractive beams, offering readers a comprehensive overview of key developments and trends within specific areas of interest. We extend a warm invitation to all our readers to explore these papers, as they undoubtedly hold valuable insights and findings that can enrich and inform research endeavors across various disciplines. Now, we will provide a comprehensive overview of each paper individually.

The first study, titled “Transferability of diffractive structure in the compression molding of chalcogenide glass” and conducted by Son et al., investigated the use of Ge_28_Sb_12_Se_60_ chalcogenide glass for the compression molding of an infrared optical lens incorporating a diffractive structure. The process began with the preparation of a mold core, achieved through ultra-precision grinding of tungsten carbide. Concurrently, a chalcogenide glass preform was carefully crafted via a polishing process, designed with a specific radius to prevent gas entrapment during the molding phase. The molding process involved subjecting the test lens to various temperature conditions, utilizing the prepared mold core and preform. Detailed analysis of the diffractive structures on both the mold core and the resulting molded lens was conducted using advanced microscopy and white light interferometry. This comparison revealed that the molding temperature significantly influenced the fidelity of the diffractive structure transfer during the chalcogenide glass lens molding process. It was found that by precisely adjusting the molding temperature, the diffractive structure of the tungsten carbide mold core could be accurately and completely replicated onto the surface of the chalcogenide glass lens. These findings highlighted the critical role of temperature control in the compression molding process for achieving high-quality diffractive optics. Optimized chalcogenide glass-based lenses demonstrated substantial potential as cost-effective yet high-performance solutions for infrared optics applications. By ensuring the precise transfer of diffractive structures, these lenses could meet stringent performance requirements, making them highly suitable for advanced optical systems.

After that, the study “Nonparaxial propagation of Bessel correlated vortex beams in free space”, which was conducted by Petrov N.I, investigated the nonparaxial propagation of partially coherent beams carrying vortices in free space by utilizing a decomposition method to break down the incident field into coherent, diffraction-free modes. Specifically, modified Bessel-correlated vortex beams with wavefront curvature were introduced to enhance the analysis. Analytical expressions were derived to characterize the intensity distribution and the degree of coherence at various propagation distances. The research provided a thorough examination of the intensity distribution evolution during beam propagation, considering a range of source parameters. This analysis illuminated how different parameters influenced the beam’s behavior over distance. Furthermore, the effects of nonparaxiality on the propagation of tightly focused coherent vortex beams were thoroughly analyzed, revealing the intricate dynamics involved in such systems. By investigating these aspects, the study offered valuable insights into the behavior of nonparaxial vortex beams, contributing to the broader understanding of advanced optical beam propagation and coherence properties. The findings have potential applications in fields where precise beam control and manipulation are crucial, such as optical communication, laser machining, and advanced imaging systems.

The study “Dividing the topological charge of a Laguerre-Gaussian beam by 2 using an off-axis Gaussian beam” conducted by Kovalev et al. theoretically proved and numerically confirmed that the topological charge (TC) of a single-ringed (zero-radial-index) Laguerre–Gaussian (LG) beam could be optically divided by two through interference with an off-axis Gaussian beam. The simple case was considered where the waist radii of both beams were identical. Their proof involved determining the positions of intensity nulls and the orders of the vortices around them (local topological charges). Analytically, it was shown that only the intensity nulls in the half-plane perpendicular to the propagation direction contributed to the TC of the superposition. Numerical simulations confirmed the theory, demonstrating the integer division of TC divided by two in simple cases. However, calculating amplitude and phase in regions of very low intensity proved difficult, preventing them from demonstrating the division of high TC values. Similar measurement challenges exist in real experiments, compounded by noise. The derivation of the TC value was only for the initial plane, and determining TC in other planes would be more complex. Upon propagation, optical vortices can move or reappear in other transverse planes, but they did not investigate these planes in this study. The author suggested that future research will explore how TC changes when the LG beam with a zero radial index is replaced by an LG beam of an arbitrary radial order, or if the Gaussian beam is replaced by another field. The behavior of optical vortices in the considered superposition upon free-space propagation also remains to be studied. The potential application area is in optical computing machines, where data are carried by vortex light beams and encoded by topological charges.

Speckle patterns were formed by random interferences of mutually coherent beams. While speckles were often considered unwanted noise in many areas, they also formed the foundation for the development of numerous speckle-based imaging, holography, and sensing technologies. In recent years, artificial speckle patterns were generated with spatially incoherent sources using static and dynamic optical modulators for advanced imaging applications. In the report “Tuning axial resolution independent of lateral resolution in a computational imaging system using Bessel Speckles” written by Anand et al., a basic study was carried out with Bessel distribution as the fundamental building block of the speckle pattern (i.e., speckle patterns formed by randomly interfering Bessel beams). In general, Bessel beams had a long focal depth, which in this scenario was counteracted by the increase in randomness, enabling tunability of the axial resolution. As a direct imaging method could not be applied when there was more than one Bessel beam, an indirect computational imaging framework was applied to study the imaging characteristics. This computational imaging process consisted of three steps. In the first step, the point spread function (PSF) was calculated, which was the speckle pattern formed by the random interferences of Bessel beams. In the next step, the intensity distribution for an object was obtained by a convolution between the PSF and object function. The object information was reconstructed by processing the PSF and the object intensity distribution using non-linear reconstruction. In the computational imaging framework, the lateral resolution remained constant, while the axial resolution improved when the randomness in the system was increased. Three-dimensional computational imaging with statistical averaging for different cases of randomness was synthetically demonstrated for two test objects located at two different distances. The presented study would lead to a new generation of incoherent imaging technologies.

In the paper “Spin-orbital conversion with the tight focus on an axial superposition of a high-order cylindrical vector beam and a beam with linear polarization” written by Kotlyar et al., a theoretical and numerical investigation was conducted on spin–orbital conversion in the tight focus of an axial superposition of a high-order (order m) cylindrical vector beam and a beam with linear polarization. Although such a beam did not have spin angular momentum in the initial plane and the third projection of its Stokes vector was equal to zero, subwavelength local regions with a transverse vortex energy flow and with a non-zero third Stokes projection (the longitudinal component of the spin angular momentum) were formed in the focal plane for an odd number *m*. This meant that such a beam with an odd *m* had regions of elliptical or circular polarization with alternating directions of rotation (clockwise and counterclockwise) in the focus. For an even *m*, the field was linearly polarized at every point of the focal plane, and the transverse energy flux was absent. These beams could be used to create a micromachine in which two microparticles in the form of gears were captured in the focus of the beam into neighboring local areas in which the energy flow rotated in different directions, and, therefore, these gears rotated in different directions.

Circular airy vortex beams (CAVBs) have garnered significant attention due to their “abruptly autofocusing” effect, phase singularity, and potential applications in optical micromanipulation, communication, and more. In the paper “Propagation characteristics of circular airy vortex beams in a uniaxial crystal along the optical axis”, Zheng et al. investigated the propagation properties of circular airy beams (CABs) imposed with different optical vortices (OVs) along the optical axis of a uniaxial crystal for the first time. Similar to other common beams, a left-hand circular polarized (LHCP) CAVB, propagating along the optical axis in a uniaxial crystal, could excite a right-hand circular polarized (RHCP) component superimposed with an on-axis vortex of a topological charge (TC) number of 2. When the incident beam was an LHCP CAB imposed with an on-axis vortex of a TC number of *l* = 1, both components exhibited an axisymmetric intensity distribution during propagation and formed hollow beams near the focal plane due to the phase singularity. The phase pattern revealed that the LHCP component carried an on-axis vortex of a TC number of *l* = 1, while the RHCP component carried an on-axis vortex of a TC number of *l* = 3. With a larger TC number (*l* = 3), the RHCP component exhibited a larger hollow region in the focal plane compared to the LHCP component. Cases of CABs imposed with one and two off-axis OVs were also studied. The off-axis OV caused the CAVB’s profile to remain asymmetric throughout the propagation. As the propagation distance increased, the off-axis OVs moved closer to the center of the beam and overlapped, resulting in a distinct intensity and phase distribution near the focal plane.

In the paper “Optical force and torque on a graphene-coated gold nanosphere by a vector Bessel beam”, Yan et al. investigated an optical force and torque on a graphene-coated gold nanosphere with a vector Bessel beam within the framework of the generalized Lorenz–Mie theory. The dielectric function of the gold core was described by the Drude–Sommerfeld mode, while that of the graphene coating was given by the Lorentz–Drude model. The coating consisted of N layers of graphene. The axial optical force and torque were numerically discussed, with particular emphasis on the effects of the graphene coating thickness (layer number N) and beam parameters, including the half-cone angle *α*_0_, order *l*, and polarizations. Numerical results showed that LSPR peaks were observed when a graphene-coated gold nanosphere was placed in a vector Bessel beam. Increasing the graphene coating thickness caused the LSPR peaks to shift towards longer wavelengths and become wider. Moreover, the increase in graphene coating thickness resulted in a smaller optical force and torque. Additionally, the LSPR peaks were highly sensitive to the beam parameters *α*_0_, order *l*, and polarizations. Therefore, by selecting appropriate beam parameters and graphene coating thickness, better manipulation, and rotation of the particle at desirable wavelengths could be achieved.

In the paper “Theoretical analysis of airy-Gauss Bullets obtained by means of high aperture Binary Micro Zonal Plate”, the methodology for obtaining vectorial three-dimensional bullets, specifically Airy–Gauss bullets, was theoretically analyzed by Blaya et al. Binary micro zonal plates (BZP) were designed to produce different Airy–Gauss bullets with sub-diffraction main lobe widths. Extending the theory beyond the electrical field, the analysis included the magnetic field. Several properties such as the Poynting vector and the energy of Airy–Gauss vectorial bullets generated by illuminating the designed BZP with temporal Gaussian circular polarized pulses were examined.

Micro-drilling transparent dielectric materials using non-diffracting beams impinging orthogonally to the sample was achieved without the need to scan the beam position along the sample thickness. In the work “Micro-hole generation by high-energy pulsed Bessel beams in different transparent materials”, Belloni et al. applied a laser micromachining process that combined picosecond pulsed Bessel beams with the trepanning technique to various transparent materials. They demonstrated the capability to create through-apertures with diameters on the order of tens of micrometers in dielectric samples that exhibited different thermal and mechanical characteristics and thicknesses, which ranged from two hundred to five hundred micrometers. The authors highlighted the advantages and drawbacks of applying this technique to materials such as glass, polymer, and diamond by analyzing the features, morphology, and aspect ratio of the generated through-holes. Additionally, they discussed alternative Bessel beam drilling configurations and explored the possibility of optimizing the quality of the aperture at the output sample/air interface, particularly in the case of glass.

Optical vortex (OV) beams were widely used to generate light fields with transverse energy flow, inducing the orbital motion of nano- and microparticles in the transverse plane. In the study “Generation of complex transverse energy flow distributions with autofocusing optical vortex beams”, Khonina et al. presented new modifications of OV beams with autofocusing properties for shaping complex transverse energy flow distributions varying in space. The angular component of the complex amplitude of these beams was defined by the superpositions of OV beams with different topological charges. This approach provided a convenient method to control the three-dimensional structure of the generated autofocusing OV beams. Controlling the transverse distribution of an autofocusing beam enabled the generation of a wide variety of fields with both rotating and periodic properties, useful in laser manipulation and laser material processing. The obtained numerical results predicted different types of motion for the trapped particles when using the designed OV autofocusing beams. Experimental results agreed with the modeling results and demonstrated the feasibility of shaping such laser beams using spatial light modulators.

The achievable resolution of a conventional imaging system was inevitably limited by diffraction. Dealing with precise imaging in scattering media, such as in biomedical imaging, was even more challenging due to weak signal-to-noise ratios. Recent developments in non-diffractive beams, including Bessel beams, Airy beams, vortex beams, and Mathieu beams, paved the way to address some of these challenges. The review titled “Bessel beams in ophthalmology: A review” and written by Sandeep et al. specifically focused on non-diffractive Bessel beams for ophthalmological applications. The review began by discussing the theoretical foundation of non-diffractive Bessel beams, followed by an analysis of various ophthalmological applications utilizing these beams. The advantages and disadvantages of these techniques were compared to those of existing state-of-the-art ophthalmological systems. The review concluded with an overview of current developments and future perspectives of non-diffractive beams in ophthalmology.

The review paper titled “Bessel beam: Significance and applications—A progressive review” and written by Khonina et al. discussed that diffraction is a phenomenon related to the wave nature of light, which arises when a propagating wave encounters an obstacle. Consequently, the wave transforms the amplitude or phase, leading to diffraction. The wavefront segments that bypass the obstacle form a diffraction pattern through mutual interference. In this review paper, they discussed the topic of non-diffractive beams, specifically Bessel beams. These beams exhibit resistance to diffraction, making them a remarkable alternative to Gaussian beams in various contexts. Numerous notable applications of Bessel beams have been identified and employed in commercial settings. The authors explored several prominent applications of these exceptional beams, including optical trapping, material processing, free-space long-distance self-healing beams, optical coherence tomography, super resolution, sharp focusing, polarization transformation, increased depth of focus, birefringence detection based on astigmatically transformed Bessel beams, and encryption in optical communication.
